# Associations between interarm differences in blood pressure and cardiovascular disease outcomes: protocol for an individual patient data meta-analysis and development of a prognostic algorithm

**DOI:** 10.1136/bmjopen-2017-016844

**Published:** 2017-07-02

**Authors:** Christopher E Clark, Kate Boddy, Fiona C Warren, Rod S Taylor, Victor Aboyans, Lyne Cloutier, Richard J McManus, Angela C Shore, John L Campbell

**Affiliations:** 1 Primary Care Research Group, Institute of Health Services Research, University of Exeter Medical School, Exeter, Devon, UK; 2 Patient and Public Involvement Team, PenCLAHRC, University of Exeter Medical School, Exeter, Devon, UK; 3 Department of Cardiology, Dupuytren University Hospital, and Inserm 1098, Tropical Neuroepidemiology, Limoges, France; 4 Département des sciences infirmières, Université du Québec à Trois-Rivières, Trois-Rivières, Canada; 5 Nuffield Department of Primary Care Health Sciences, University of Oxford, Oxford, UK; 6 NIHR Exeter Clinical Research Facility, Royal Devon and Exeter Hospital and University of Exeter Medical School, Exeter, Devon, UK

**Keywords:** PRIMARY CARE, Hypertension, Ischaemic heart disease, VASCULAR MEDICINE, STROKE MEDICINE

## Abstract

**Introduction:**

Individual cohort studies in various populations and study-level meta-analyses have shown interarm differences (IAD) in blood pressure to be associated with increased cardiovascular and all-cause mortality. However, key questions remain, such as follows: (1) What is the additional contribution of IAD to prognostic risk estimation for cardiovascular and all-cause mortality? (2) What is the minimum cut-off value for IAD that defines elevated risk? (3) Is there a prognostic value of IAD and do different methods of IAD measurement impact on the prognostic value of IAD? We aim to address these questions by conducting an individual patient data (IPD) meta-analysis.

**Methods and analysis:**

This study will identify prospective cohort studies that measured blood pressure in both arms during recruitment, and invite authors to contribute IPD datasets to this collaboration. All patient data received will be combined into a single dataset. Using one-stage meta-analysis, we will undertake multivariable time-to-event regression modelling, with the aim of developing a new prognostic model for cardiovascular risk estimation that includes IAD. We will explore variations in risk contribution of IAD across predefined population subgroups (eg, hypertensives, diabetics), establish the lower limit of IAD that is associated with additional cardiovascular risk and assess the impact of different methods of IAD measurement on risk prediction.

**Ethics and dissemination:**

This study will not include any patient identifiable data. Included datasets will already have ethical approval and consent from their sponsors. Findings will be presented to international conferences and published in peer reviewed journals, and we have a comprehensive dissemination strategy in place with integrated patient and public involvement.

**PROSPERO registration number:**

CRD42015031227.

**Strengths and limitations of this study**

- This individual participant data (IPD) meta-analysis brings together the largest dataset yet assembled to study the association of interarm blood pressure differences with mortality and morbidity.
- IPD permits a consistent approach to all of the available data that cannot be achieved with study-level meta-analyses. It maximises statistical power to allow a full exploration of the prognostic association between interarm differences and time-to-event outcomes.
- Patient and public involvement is embedded at every step of this study.
- Inclusion of cohorts from around the world will maximise the ability to generalise our findings.
- We will have sufficient data to explore subgroup and sensitivity analyses addressing questions that cannot be answered at individual study level or through aggregated meta-analyses.
- Data collection, including methods of blood pressure measurement, varies between cohorts and is an acknowedged limitation of the data; this will be addressed in planned senstivity analyses.

## Introduction

Cardiovascular disease (CVD) is the leading cause of death worldwide. Elevated blood pressure (BP) is the main global risk factor for premature morbidity and mortality, and the prevalence of hypertensive heart disease is not declining over time.^1 2^ Control of high BP is therefore fundamental in the prevention of CVD.^3^ Prevention of CVD is promoted by the UK Quality and Outcomes Framework, within which BP control is a key indicator,^3^ and BP measurement is a common reason for consultation in primary care.^4^ Although the benefits of treatment in hypertension are greatest for individuals at the highest estimated risk,^5^ the majority of events occur in those at low to medium risk.^6^ Therefore, recognition of novel CVD risk markers to refine risk prediction and stratify treatment priorities is important.^7 8^

A systolic BP difference between arms (interarm difference, IAD) is one risk marker that is easily measured clinically with no additional equipment. Differences between arms can cause errors in BP interpretation and management when not recognised.^9–13^ A systolic IAD is regularly encountered in clinical practice: in community settings, differences ≥10 mm Hg in systolic pressures are seen in 11.2% (95% CI: 9.1% to 13.6%) of hypertensive subjects, 7.4% (5.8% to 9.2%) of people with diabetes and 3.6% (2.3% to 5.0%) of the general population.^14^ Prevalence rates are higher in outpatient and hospital settings;^15^ thus, questions about the importance of a finding of IAD frequently arise.^16^ Recommendations to initially check BP in both arms are included in international hypertension guidelines,^17–21^ but may not followed by the majority of clinicians,^22 23^ including UK general practitioners, due to a lack of clarity as to the importance of detecting an IAD.^24 25^

IAD can cause errors in BP interpretation and management when not recognised, thus exposing individuals to avoidable risk through suboptimal BP control.^9–13^ In individual studies, systolic IADs are associated in cross-sectional analyses with greater prevalence of CVD,^26 27^ peripheral arterial disease,^28^ cerebrovascular disease,^28 29^ diabetes^30–32^ and hypertension.^32 33^ Study-level meta-analyses, however, disagree concerning associations in hypertension and diabetes.^15 16^ IADs are also independently associated with increased left ventricular mass,^34^ arterial stiffness^30 34 35^ and in diabetes with nephropathy^31 36^ and retinopathy.^31^ Recent data suggest that IAD may also be associated prospectively with greater cognitive decline.^37 38^

Prospectively, systolic IADs are associated with elevated cardiovascular and all-cause mortality, and with increased CVD event rates.^29 31 32 39–46^ Evidence for this association is derived from a number of cohort studies and our 2012 meta-analysis of study-level aggregate data.^28^ Since our systematic review and meta-analysis, several new large cohort studies have been published that include patients with diabetes, those at CVD risk and general populations. We and others have updated study-level analyses to include these;^47 48^ however, this approach is limited in the conclusions that can be drawn, because it combines studies with different patient characteristics, different methodological choices (eg, in choice of cut-off values for IAD) and different analytical approaches. By obtaining the original individual participant data (IPD) from these cohorts, a consistent approach to all of the data will, for the first time, provide the necessary information and maximise statistical power to allow a full exploration of the prognostic association between systolic IAD and time-to-event outcomes across populations of differing baseline CVD risk, and allow adjustment for important confounders in a standardised manner.

This study will undertake an IPD meta-analysis to address the following research questions.

## What is the additional contribution of systolic IAD to prognostic risk estimation for cardiovascular fatal and non-fatal events, and all-cause mortality?

By taking account of a systolic IAD, the precision of cardiovascular risk prediction is increased when compared with that achieved by using the Framingham model alone,^42 46^ in a similar manner to that shown for the ankle–brachial index.^49^ However, individual studies use different models of adjustment for other known cardiovascular risk factors, which limits our ability to definitively address this question with aggregate study-level data alone. HRs associated with systolic IADs are greater in cohorts with high background CVD risk in comparison with cohorts representative of primary care populations,^48 50^ suggesting that the prognostic contribution of systolic IAD to risk prediction varies with underlying population cardiovascular risk.

## What is the cut-off value for IAD that defines elevated risk and is there a relationship between increasing IAD and increasing risk?

Survival differences have been demonstrated for systolic IADs ≥5, 10 and 15 mm Hg,^43 45^ but no consistent relationship between higher systolic IADs and greater HRs has yet emerged.^47 48^ Consequently, uncertainty remains as to the appropriate cut-off level of systolic IAD for risk prediction.^28 41 51^ Further investigation is required to determine what, if any, systolic or diastolic IAD cut-off can be regarded as clinically important.

## Do different methods of IAD measurement have an impact on the prognostic value of IAD measurements?

In clinical practice, most people measure IAD sequentially because they only have access to one single-cuff BP measurement device. There are some data suggesting that simultaneous measurement of both arms at once is more accurate, in prognostic terms, than sequential measurement.^52 53^ This project will evaluate whether different measurement methods influence the prognostic impact of IAD, and inform the future recommendations for the measurement of IAD.

## Methods and analysis

### Aims and objectives

This IPD meta-analysis has the following aims.

1. To undertake an updated systematic review with comprehensive literature searches to identify any hitherto unknown potentially eligible relevant IAD datasets and invite their chief investigators to join the collaboration. Searches will run and be updated to January 2017.
2. To standardise and combine IPD from prospective observational cohorts that measured BP in both arms during patient recruitment, and which collected morbidity and mortality outcomes in adult populations.
3. To develop overarching (across studies) multivariable time-to-event models for all-cause mortality, CVD mortality, non-fatal CVD events, risk for fatal and/or non-fatal cardiovascular events, and for all-cause mortality, to establish any additional contribution of systolic IAD to risk prediction. We aim to use these results in inform the development of a new prognostic model of CVD risk estimation that takes account of systolic IAD.
4. To examine the prospective univariable and multivariable associations of systolic IAD with any change in cognitive function.
5. Explore any additional risk contribution of systolic IAD across predefined subgroups based on underlying health status (ie, with or without existing CVD, diabetes or hypertension), when adjusted for major confounders such as gender, age or ethnicity.
6. Explore the lower limit of magnitude for a systolic IAD, at which the IAD adds significantly to the risk prediction model, to define a clinically important cut-off value for IAD based on prognostic value.
7. Undertake a subgroup analysis by method of IAD measurement (ie, sequentially vs simultaneously measured BPs) to explore any effect of different methods on risk prediction by IAD.
8. Undertake cross-sectional analyses to describe the epidemiology of IAD in the dataset. We will look for variations in the prevalence of IAD by age, gender and ethnicity, and we will describe the risk factors or disease conditions associated with the presence of an IAD. This information can inform guidance as to whom we should assess for the possible presence of IAD in the future.

### Search strategy for identification of studies

Relevant electronic databases (Medline, Old Medline, Medline in Process, Embase and CINAHL) will be searched for all articles published since their respective start dates that potentially meet the inclusion criteria (see online appendix: Medline search strategy). The search strategy will be verified through scoping searches checking for inclusion of all potentially relevant cohorts already known to the authors. Searches will be augmented by contacts with authors active in the field, and by dissemination of our interest through established cardiovascular intervention collaborations. Regular update searches will be run during the project.

10.1136/bmjopen-2017-016844.supp1 Supplementary data 1

### Eligibility criteria for studies

Studies will be eligible for inclusion in the IPD dataset if they meet the following criteria.

- *Design:* prospective cohort studies.
- *Population:* adults aged 18 or over, with or without existing CVD, with a record of BP in both arms at recruitment. BP must have been measured in both arms during the same assessment but datasets based on either simultaneous or sequential methods of measurement will be included.
- *Setting:* community or primary care cohorts or (to account for varied healthcare systems) based in hospital clinic settings relevant to a wider population, for example, unselected diabetes or hypertension cohorts. Selected secondary or tertiary care cohorts such as those with renal disease or attending vascular disease clinics will be excluded.
- *Sample size:* no minimum sample size will be defined.
- *Publication language and date*: no restriction.
- *Outcome*: CVD mortality or all-cause mortality, fatal and non-fatal CVD events, or measures of cognitive decline.

### Assessment

Study selections will be undertaken by two authors independently. Disagreements will be resolved by discussion when possible, and failing that adjudication from the remaining reviewing authors will be sought. The quality of included cohorts will also be assessed using the Quality Improvement in Prognostic Studies (QUIPS) tool.^54^

### Data collection

We will ask study authors to provide the following data (if available):

#### Study-level descriptive data

- Setting: community, primary care or hospital cohort.
- Population: selected, for example hypertension, or general.
- Method of BP measurement: monitor used, number of readings, position (seated or supine).
- Method of BP and IAD measurement: sequential or simultaneous; any rounding of readings.

#### Participant-level descriptive data

- Demographic details: age, sex, ethnicity, height and weight (or body mass index), dominant hand.
- Geographical location of partcipants.
- Medical history: specifically of cardiovascular or peripheral arterial disease, cerebrovascular disease, cardiac arrhythmias (diabetes or chronic kidney disease).
- Smoking history.
- Medications prescribed at baseline.
- Individual baseline BP readings for each arm and heart rate (or means if individual readings are not available).
- Biochemical measurements: glucose or glycosylated haemoglobin, total and high density lipoprotein (HDL) cholesterol (fasting or non-fasting), estimated glomerular filtration rate, creatine and uric acid.
- Measures of CVD risk: for example, Framingham score, Qrisk2 score.^55 56^
- Ankle–brachial index.
- Any measures or scores reporting deprivation.
- Any baseline measures of cognition, for example, Mini Mental State Examination.
- Highest level of educational attainment.

#### Participant-level outcome data

- We will seek the number and timings of the following events: all-cause deaths, cardiovascular deaths, non-fatal cardiovascular events, any outcome assessments of cognition.

### Data transfer and cleaning

Data will be transferred by the study teams in line with University of Exeter Standard Operating Procedures covering electronic transfer.^57^

On successful transfer, the data will be imported into Microsoft SQL server as a table or table set per study for cleaning. We are requesting that data are anonymised before transfer; should this not happen, anonymisation will occur before the backup run (midnight) on the day of import to prevent identifiable information entering the backup sets and requiring further action. The table/table sets for each study will remain separate throughout the cleaning process, maintaining data integrity prior to combining the study-level datasets, and will be versioned so a history of the cleaning operations can be reviewed as needed, in co-ordination with the statistician.

Finally, the datasets will be joined into a single live view/table for statistical analysis, preferably via direct connection of a statistical software package to SQL server, though static exported files can also be created.

Access to data at all stages of cleaning and analysis will be restricted to specific members of staff and secured via permissions-dependant user logins, and again follow the Standard Operating Procedures if export/transfer outside of the SQL server system is required.

### Statistical analysis

#### Descriptive analysis and data checking

Study-level data on study and patient characteristics of included IPD studies will be summarised in detail, and compared with data published in the original study publication, if available. Comparison with study and patient characteristics (where available) of cohort studies that do not contribute data to the IPD analysis will be made to descriptively assess study inclusion bias.

IAD will be calculated as the absolute difference between systolic BP in the right and left arms. If multiple values for BP are provided, the mean of the observations will be used. For each study, the mean IAD and proportions of patients with an IAD equal to or exceeding the three commonly reported cut-off values (5, 10 and 15 mm Hg) will be reported. We will note the number of studies that include the necessary variables for calculation of Framingham,^55^ QRisk2^56^ and other scores as emerge from the literature searches. If necessary, we will standardise covariates that vary across individual studies to permit incorporation of that data across studies.

#### Individual patient data meta-analysis

The outcomes to be investigated are (1) time to CVD mortality; (2) time to all-cause mortality; and (3) time to CVD morbidity. All analyses will be performed for each of these three outcome variables. We will undertake both one-stage and two-stage meta-analyses as follows; however, the focus will be on the one-stage meta-analysis. Initial two-stage models will be performed using combined data from all datasets, for all outcomes at 3, 5 and 10-year follow-up cut-off points, using study-level outcomes adjustment for baseline systolic BP, age and gender. Such models will be used for comparison with the results of equivalent one-stage models, and to estimate the I-squared heterogeneity statistic.^58 59^

For the one-stage models, we will non-randomly select a subset of the datasets available to act as a validation dataset; the selected datasets will include participants of both genders and a range of ages.^60^ To account for likely statistical heterogeneity of the datasets (investigated by two-stage models), we anticipate using random effects time to event analysis methods. We will aim to use random effects methods with a flexible parametric survival model.^61 62^ Alternative parametric models will be considered if the dataset is amenable to such models. An initial series of models will investigate the association between IAD and outcome; IAD will be included as a random effect (allowed to vary within study) in all models, and all models will include individual study as a fixed effect. A series of models will investigate whether other patient-level covariates are also significant predictors of outcome, with inclusion of IAD within the model. Each individual covariate will be investigated by inclusion as a fixed effect in each model with only IAD and study. In a further model, all covariates will be included. Any covariates that are statistically significant (at the p<0.1 threshold) will be included in a further model, and only those with a p value<0.05 will be included in the model. Each non-included covariate will then be included individually to identify whether addition of the covariate improves model fit. This process will allow us to develop a model that maximises goodness of fit with the fewest statistically significant covariates. This model will be compared with the Framingham set of covariates (essential covariates; table 1). If the models include different covariates, we will compare the goodness of fit for our model with that of the Framingham model. We will also repeat this approach with the inclusion of study-level covariates (setting, method of measurement, temporal sequence of measurement, geographical region). The Akaike Information Criterion^63^ will be used to assist in model selection.

**Table 1:** Essential and desirable patient-level covariates for meta-analysis identified a priori

	Study	Thomas *et al* 38 73	Clark and Powell^39^	Aboyans *et al* 40	Agarwal *et al* 41	Clark *et al* 42	Clark *et al* 43	Kim *et al* 29	Sheng *et al* 32	Durrand *et al* 44	White *et al* 45	Clark *et al* 31	Weinberg *et al* 46
Suggested essential minimum dataset for analyses	Age	X	X	X	X	X	X	X	X	X	X	X	X
	Gender	X	X	X	X	X	X	X	X	X	X	X	X
	BMI	X	X	X	X	X		X	X		X	X	X
	Smoking	X	X	X	X	X	X	X	X		X	X	X
	Systolic BP	X	X	X	X	X	X		X		X	X	X
	Diastolic BP	X	X		X	X	X		X		X	X	X
	Hypertensive Yes/no	X	X	X	X	X		X	X	X	X	X	X
	Lipids (total and HDL)	X	X	X		X	X	X	X		X	X	X
	Diabetic Yes/no	X		X	X	X	X	X	X	X	X	X	X
Desirable further data for sensitivity analyses	HbA1c	X										X	
	Chronic Kidney Disease (CKD) Yes/no	X			X					X		X	
	Presence of CKD, record of estimated glomerular filtration rate or creatine	X			X			X (creatine)				X	
	Ankle–brachial index			X			X		X		X		
	Framingham score					X							X
	Ethnic grouping	X	X	X		X	X	X	X		X	X	X

BMI, body mass index; BP, blood pressure.

A series of models will then be performed using binarised IAD, with cut-off points ranging from 0 to 20 mm Hg, with increments of 1 mm Hg.^64 65^ The set of covariates previously derived will be used with the aim of establishing a cut-off point at which IAD becomes a significant predictor of risk.

A further issue of interest is whether there are any differential effects of IAD across different patient-level characteristics, for example, age, gender and baseline systolic BP. To investigate possible differential effects, we will perform a series of models, using the model including significant predictors previously developed. Each model will include the specified covariate and its interaction term with IAD, as well as the other patient-level characteristics. Interactions will be considered significant at the p value<0.05, and interpreted in the light of multiple testing, although we acknowledge that power to detect interaction terms will be limited. We will also investigate whether the effect of IAD varies with time by including an interaction between time and IAD. Finally, we will investigate whether there is an interaction effect between measurement method of IAD (simultaneous or sequential) and IAD by adding measurement method and its interaction with IAD into the model including all significant predictors of outcome. We will use appropriate centring of variables within each study for the interaction models.

#### Missing data

The percentage of individual participant missing data will be reported for each study and participant-level covariate. Baseline covariates will be included in the modelling if the covariate has at least 50% data present in at least three studies.^66^ Imputation of participant-level missing baseline data across the whole dataset will be performed; the imputation model will take account of individual study. The effect of inclusion of imputed data will be checked using a fixed effect model including only the imputed covariate (as well as IAD). Analyses including imputed data will be considered as sensitivity analyses only. The number of studies with study-level missing baseline data (ie, no data for a specified variable was collected within the study) will be reported for each variable. The course of action of imputing missing data at the study level will be considered should the number of studies with study-level missing baseline data be substantive for individual covariates.

#### Model validation

The final model will be validated using the calibration slope method,^67^ and internal–external cross validation analysis.^68 69^ Should the prognostic model be found to perform poorly for any outcome, we will consider possible reasons for such poor performance. It may be appropriate to develop a model that is applicable only to a subset of studies, for example, including studies from the same clinical setting or in similar patient populations.

#### Incorporation of IAD into a prognostic model based on the Framingham score

We will aim to incorporate IAD (if found to be a significant prognostic factor for mortality/morbidity) into the Framingham scoring system for predicting cardiovascular risk. This model will be developed using the overall dataset including all studies that include the Framingham covariates. Calibration slopes comparing the observed and expected survival probabilities at different time points will be calculated, and combined in a two-stage meta-analysis for each approach. The pooled estimates from these models will be used to compare the calibration for each model.

#### Publication and inclusion bias

We will undertake two-stage analyses to compare aggregate level data from contributing and non-contributing datasets to test for selection bias. We will qualitatively assess inclusion bias by comparing study-level data on key variables (eg, age, gender and BP) for cohorts that meet our inclusion criteria but for which consent to share data is not obtained, with our IPD dataset. We will also check whether our collection of studies may be affected by publication bias using a funnel plot and Egger’s test for each of the outcome variables to confirm asymmetry.^70 71^

#### Quality assessment

We will assess individual study quality using the QUIPS tool.^54^ Based on the variation in quality observed, we will consider restriction of the models to higher quality studies, using criteria to be determined.

## Patient and public involvement

The development of this protocol has had considerable patient and public involvement (PPI) input. Prior to funding, a draft was reviewed by three separate user involvement groups improving the overall clarity in general, and in specific areas such as focussing the research questions on aspects of IAD that interest users. We convened two prefunding public workshops to raise awareness about involvement in systematic reviews and gain critical feedback for the project. This feedback resulted in a clearer definition of the population being studied, greater clarity about benefits for patients and reinforcement of our user dissemination plans.

We have established a PPI group, specifically for the project, who will play an important role in shaping the research by taking part in the bimonthly management meetings. The group has contributed towards the drafting of the protocol. We plan two key workshops to ensure that the review findings reach the end user in an accessible way. First, a summary writing workshop with the PPI group to achieve a clear plain language summary and to coproduce a dissemination plan targeted at patients and the public. Second, we will convene a larger public event on the subject of understanding cardiovascular risk, within which the findings of this research can be presented in context.

## Ethics and dissemination

This is a secondary analysis of patient anonymised data. All data will have been obtained from studies where patients will have already given their consent and approval to participate. We will seek written permission for use of individual patient data from each individual study lead investigator. We will therefore not seek no further ethical approval.

The study will be reported in accordance with the Preferred Reporting Items for a Systematic Reviewand Meta-analysis of Individual Participant Data (PRISMA-IPD) statement.^72^ Findings will be presented at international conferences and published as open access articles in high-impact journals. Through targeted briefings, we will seek to inform authoritative national, European and global developers of clinical guidelines including the British Hypertension Society, UK National Institute for Health and Care Excellence and NHS commissioners and providers locally. We will produce a targeted dissemination plan for the public in conjunction with the project’s PPI group. We will specifically target local patient participation groups and relevant hypertension charities. We will also plan a public dissemination event for patients, clinicians and providers or commissioners on the subject of understanding cardiovascular risk, at which these findings will be presented.

The INTERPRESS collaboration will act as an international forum for clinicians and researchers with an interest in IAD and act as a future platform for international research activity in this area.

## Discussion

The results of INTERPRESS IPD meta-analysis will inform the practice of clinicians who need to manage a patient with an IAD. Our findings will define populations with small IADs who can be reassured over this clinical finding, and provide the evidence to identify those with clinically important IADs who may benefit either individually from further cardiovascular investigation or at a population level through further research for interventions to mitigate risk. Our results will help clinicians to inform patients about their future risk more accurately. Patients will benefit individually by being better informed about any excess CVD risk based on their interarm BP differences.

Although our previous findings have already contributed to international clinical guidelines on BP measurement,^21 28^ statements in current versions are still largely based on expert opinion rather than evidence. INTERPRESS will provide important new evidence that will directly inform future updates of international guidelines and clinical practice and impact on patient care.

## Supplementary Material

Reviewer comments
