# Supplementary material for: Associations between interarm differences in blood pressure and cardiovascular disease outcomes: protocol for an individual patient data meta-analysis and development of a prognostic algorithm
Source: BMJ Open. 2017 Jul 2;7(6):e016844. doi: 10.1136/bmjopen-2017-016844 (PMC5734572; doi:10.1136/bmjopen-2017-016844)
Supplement: Supplementary data 1 [file bmjopen-2017-016844supp001.pdf]

Appendix – Medline Search strategy (adapted for use in Embase and CiNAHL)

No	Search term
1	subclavian.ti,ab.
2	stenosis.ti,ab.
3	1 and 2
4	inter-arm.ti,ab.
5	interarm.ti,ab.
6	4 or 5
7	blood pressure*.ti.
8	differen*.ti.
9	7 and 8
10	ABI.ti,ab.
11	ABPI.ti,ab.
12	ankle brachial pressure.ti,ab.
13	ankle brachial pressure index.ti,ab.
14	10 or 11 or 12 or 13
15	3 or 6 or 9 or 14
16	exp Cohort Studies/
17	cohort.mp.
18	follow up.mp.
19	16 or 17 or 18
20	15 and 19
