# Supplementary material for: Associations between interarm differences in blood pressure and cardiovascular disease outcomes: protocol for an individual patient data meta-analysis and development of a prognostic algorithm
Source: BMJ Open. 2017 Jul 2;7(6):e016844. doi: 10.1136/bmjopen-2017-016844 (PMC5734572; doi:10.1136/bmjopen-2017-016844)
Supplement: Reviewer comments [file bmjopen-2017-016844.reviewer_comments.pdf]

PEER REVIEW HISTORY

BMJ Open publishes all reviews undertaken for accepted manuscripts. Reviewers are asked to complete a checklist review form (**http://bmjopen.bmj.com/site/about/resources/checklist.pdf**) and are provided with free text boxes to elaborate on their assessment. These free text comments are reproduced below.

ARTICLE DETAILS

**TITLE (PROVISIONAL)**	Associations between inter-arm differences in blood pressure and cardiovascular disease outcomes: Protocol for an individual patient data meta-analysis and development of a prognostic algorithm
**AUTHORS**	Clark, CE; Boddy, Kate; Warren, Fiona; Taylor, Rod; Aboyans, Victor; Cloutier, Lyne; McManus, Richard; Shore, Angela; Campbell, John

This article was not externally reviewed at BMJ Open. Protocols that have been independently assessed prior to submission to BMJ Open are usually fast-tracked to publication on the grounds that further substantial changes will not be possible. This independent assessment will usually be external, independent review for both a substantial grant award from a non-commercial or government funder and ethics approval.
